# Outbreak of Shiga toxin-producing *Escherichia coli* O157:H7 linked to raw drinking milk resolved by rapid application of advanced pathogen characterisation methods, England, August to October 2017

**DOI:** 10.2807/1560-7917.ES.2019.24.16.1800191

**Published:** 2019-04-18

**Authors:** Juli Treacy, Claire Jenkins, Karthik Paranthaman, Frieda Jorgensen, Doris Mueller-Doblies, Muna Anjum, Lukeki Kaindama, Hassan Hartman, Miranda Kirchner, Therese Carson, Ishani Kar-Purkayastha

**Affiliations:** 1 Public Health England South East, Hampshire and Isle of Wight Health Protection Team, Fareham, United Kingdom; 2 National Infection Service, Public Health England, London, United Kingdom; 3 National Infection Service, Field Epidemiology Service, Public Health England, London, United Kingdom; 4 National Infection Service, Food Water and Environmental Microbiology Laboratory, Public Health England, Porton, United Kingdom; 5 Animal and Plant Health Agency, Surrey, United Kingdom; 6 Gastrointestinal Emerging and Zoonoses Infections, Public Health England, London, United Kingdom

**Keywords:** Escherichia coli O157, gastrointestinal disease, outbreaks, raw drinking milk, whole genome sequencing, England, outbreaks

## Abstract

An outbreak of Shiga toxin-producing *Escherichia coli* (STEC) O157:H7 occurred on the Isle of Wight between August and October 2017. Of the seven cases linked to the outbreak, five were identified through the statutory notification process and two were identified through national surveillance of whole genome sequencing data. Enhanced surveillance questionnaires established a common link to a farm, and link to the likely food vehicle, raw drinking milk (RDM). Microbiological investigations, including PCR, identified the presence of STEC O157:H7 in samples of RDM. Analysis of core genome single nucleotide polymorphism (SNP) data of STEC O157:H7 from human stool specimens, animal faecal samples and RDM demonstrated a one SNP difference between isolates, and therefore close genetic relatedness. Control measures that were put in place included suspension of sales and recall of RDM, as well as restrictions on public access to parts of the farm. Successful integration of traditional epidemiological surveillance and advanced laboratory methods for the detection and characterisation of STEC O157:H7 from human, animal and environmental samples enabled prompt identification of the outbreak vehicle and provided evidence to support the outbreak control team’s decision-making, leading to implementation of effective control measures in a timely manner.

## Background

Shiga toxin-producing *Escherichia coli* (STEC) belong to a group of zoonotic pathogens transmitted to humans by direct contact with contaminated food, animals or their environments, or by secondary person-to-person spread, particularly in family groups within households [1]. The natural reservoir of STEC is the gastrointestinal tract of ruminant animals; in the United Kingdom (UK), the predominant animal reservoirs are cattle and sheep. STEC cause a wide spectrum of illness ranging from mild to severe bloody diarrhoea. Haemolytic uraemic syndrome (HUS), a complication of STEC, develops in 5–15% of cases, depending on the age and sex of the case [2].

STEC are defined by the presence of the Shiga toxin-encoding genes *stx1* and *stx2*, which can be divided into subtypes *stx1a-1d* and *stx2a-2g* [3]. STEC can be classified into serotypes, with STEC O157:H7 being the commonest STEC serotype associated with human disease in England, with 600–800 cases reported annually [1].

STEC O157:H7 emerged as a pathogen of public health concern in the 1980s, when it was found to be the cause of HUS outbreaks in children [4]. To counter this threat to public health, epidemiological and microbiological surveillance systems were put in place in the UK in the 1990s, and continue to be developed and enhanced [5]. In 2009, the National Enhanced STEC Surveillance System (NESSS) was implemented at Public Health England (PHE) to collect epidemiological data on every case of STEC O157:H7 in England and Wales [1]. Isolates of STEC O157:H7 detected at local diagnostic laboratories are referred to the Gastrointestinal Bacteria Reference Unit (GBRU) at PHE for confirmatory testing and typing. Since 2015, whole genome sequencing (WGS) has been employed on all STEC submitted to GBRU to provide highly discriminatory typing for public health surveillance and to facilitate outbreak detection and investigation [6].

Food-borne outbreaks of STEC O157:H7 are difficult to detect and investigate, as they often involve a small number of geographically dispersed cases [7]. Studies have shown that the use of WGS for routine surveillance of STEC O157:H7 is a robust approach for the identification of geographically and temporally distinct cases sharing common exposures [6].

### Outbreak detection

In the outbreak described here, the first two cases—who were both residents of the Isle of Wight, off the south coast of England—were notified to PHE by local microbiologists on detection of presumptive *E. coli* O157:H7 in stool samples. A common epidemiological link between the cases and a local farm was established on 25 September 2017, and an outbreak control team (OCT) meeting convened later the same day. Just before the OCT meeting, a further case with an isolate genetically identical to the first notified case was identified through routine review of the PHE WGS database. 

This report summarises the key findings, actions and conclusions of the multi-faceted outbreak investigation, and aims to highlight the role of advanced laboratory tools such as PCR and WGS in informing a public health response.

## Methods

### Case ascertainment by enhanced epidemiological surveillance

Presumptive cases of STEC were reported directly to PHE centres by clinical microbiologists at local hospital laboratories and a standardised STEC Enhanced Surveillance Questionnaire (STEC ESQ) (https://www.gov.uk/government/uploads/system/uploads/attachment_data/file/323423/VTEC_Questionnaire.pdf) was administered to each case either by local health protection professionals or environmental health officers (EHOs). Data from the questionnaires were uploaded to NESSS. NESSS was reviewed to identify any cases with an epidemiological link to the Isle of Wight that had been notified nationally since the beginning of January 2017. Any cases identified this way, or as having a microbiological link through STEC WGS surveillance processes, were reviewed against the outbreak case definitions.

For the purposes of the outbreak investigation, a confirmed case was defined either as (i) a case with STEC O157:H7 PT21/28 with a single nucleotide polymorphism (SNP) profile within 5 SNPs of the outbreak strain profile and reported onset of symptoms since August 2017 or (ii) a case with HUS and serum antibodies to the lipopolysaccharide of *E. coli* O157 or a faecal specimen that tested positive for the *stx* gene by PCR, notified since August 2017 with a known epidemiological link to the implicated farm. Known epidemiological links included consumption of raw drinking milk (RDM) produced at the farm or being an employee or close contact of an employee at the farm. A probable case was defined as a case with HUS with a known epidemiological link to the farm, in the absence of a positive STEC microbiological result.

### Microbiological examination of food samples

Samples of RDM from the implicated farm—three from the bulk tank and six from packaged bottles—were taken by EHOs on 25 September 2017. Samples of pasteurised whole, semi-skimmed and skimmed milk from the holding tanks and from bottles were also obtained from the farm by EHOs. All samples were collected and transported in accordance with the Food Standards Agency Food Law Code of Practice (https://www.food.gov.uk/enforcement/codes-of-practice/food-law-code-of-practice-2015). They were transported to the PHE Food, Water and Environmental (FWE) Microbiology Laboratory, Porton, in cold boxes (temperature 0–8 °C) and tested within 24 hours of collection.

Tests for the detection of STEC (including STEC O157:H7), *Salmonella* species, *Campylobacter* species and *Listeria* species were performed on 25 mL samples of milk. Enumeration of coliform bacteria, *E. coli*, coagulase-positive staphylococci, aerobic colony count and *Listeria* species (including *L. monocytogenes*) was carried out using dilutions of milk samples [8]. Real-time PCR was used to examine samples for the presence of STEC O157 based on CEN/ISO TS 13136, as described previously [9]. Enrichment broths that were PCR positive for *stx* were sub-cultured onto MacConkey agar and cefixime tellurite sorbitol MacConkey agar, and up to 50 colonies were retested using the same PCR assay.

### Veterinary investigation and microbiological examination of animal faecal specimens

A Veterinary Investigation Officer (VIO) from the Animal and Plant Health Agency (APHA) visited the implicated farm on 5 October 2017 to assess the potential role of the farm’s animals as the source of infection, and to review if there were appropriate controls in place to reduce the risk of contamination of RDM from the farm environment. Faecal samples were collected from calves and cows, and were tested using immuno-magnetic separation culture methodology, as described by Pritchard et al. [10].

### Dairy hygiene inspection

The Food Standards Agency (FSA) Dairy Hygiene Inspectorate (DHI) carried out independent environmental inspections at the farm, focusing on raw milk production activity. This included collection of further raw milk samples to establish whether there was ongoing contamination of the RDM. These samples were also sent to the PHE FWE laboratory for testing.

### Molecular typing of STEC O157:H7 by whole genome sequencing

Isolates of STEC O157:H7 from clinical specimens, food and animal samples were sent to PHE GBRU for confirmation, phage typing and WGS [6,11]. DNA was extracted from cultures for sequencing on the HiSeq 2500 instrument (Illumina, California, USA). High quality Illumina reads were mapped to the STEC O157:H7 reference genome Sakai (GenBank accession BA000007) using BWA-MEM [12]. SNPs were identified using GATK2 [13] in unified genotyper mode. Core genome positions that had a high quality SNP (> 90% consensus, minimum depth 10×, GQ ≥ 30) in at least one isolate were extracted. SNP positions that were present in at least 80% of isolates were used to derive maximum likelihood phylogenies with RaxML [14] using the GTRCAT model with 1,000 iterations.

Genomes were compared with the sequences held in the PHE STEC O157:H7 WGS database. This database comprises genomes from more than 2,500 cultures of STEC O157:H7 submitted to GBRU between1982–2017. Hierarchical single linkage clustering was performed on the pairwise SNP difference between all isolates at various distance thresholds (Δ250, Δ100, Δ50, Δ25, Δ10, Δ5, Δ0). The result of the clustering is a SNP address that can be used to describe the population structure based on clonal groups. Clusters at the zero, five and 10 SNP level are highlighted for further investigation and are analysed in the context of their nearest neighbours. Isolates of STEC O157:H7 with less than five SNPs differences within their core genome were considered closely related and likely to have an epidemiological link [6]. *stx* subtyping was performed, as described by Ashton et al. [15].

FASTQ reads from all sequences in this study and the PHE STEC O157:H7 WGS data can be found at the PHE Pathogens BioProject at the National Center for Biotechnology Information (Accession PRJNA248792).

### Ethical statement

The planning, conduct and reporting of this study was in line with the Declaration of Helsinki, as revised in 2013.

## Results

### Descriptive epidemiology

**Figure 1:**
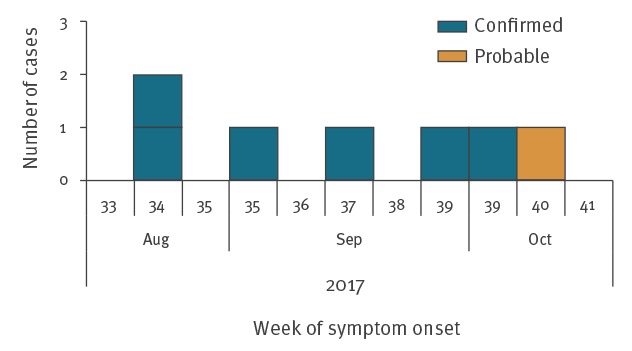
Confirmed and probable cases in STEC O157:H7 outbreak by week of symptom onset, England, 2017 (n = 7)

**Figure 2:**
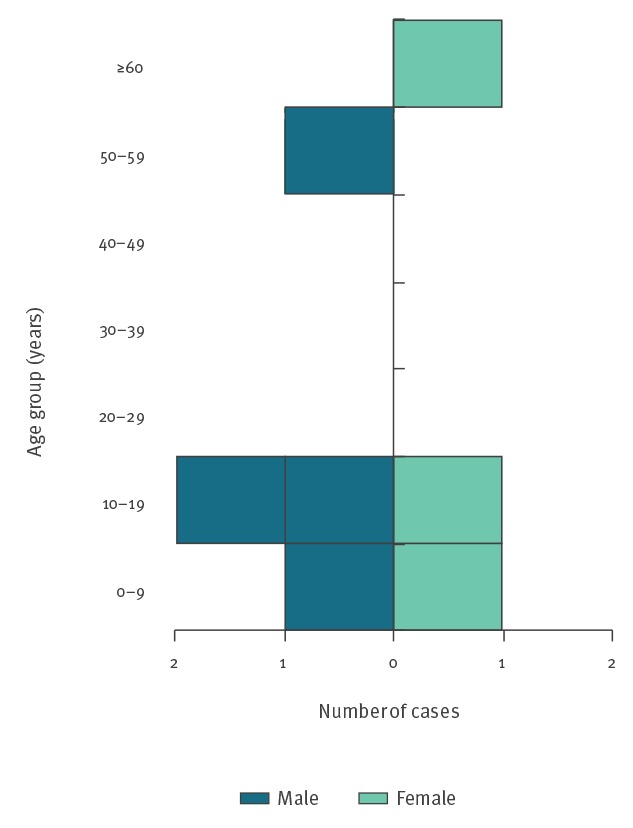
Confirmed and probable cases in STEC O157:H7 outbreak by age and sex, England, 2017 (n = 7)^a^

A total of seven cases (six confirmed and one probable) were identified as part of this outbreak, with onset of symptoms between August–October 2017 (Figure 1). STEC O157:H7 was isolated from the six confirmed cases. The probable case had HUS and drank RDM from the implicated farm, but did not provide any specimens for microbiological tests. Four of the cases were male and three were female. The median age was 10 years (range: 1–62 years; mean: 21 years) (Figure 2). The duration of symptoms ranged from 2–17 days (mean: 7 days; median: 6 days).

STEC: Shiga toxin-producing *Escherichia coli.*

STEC: Shiga toxin-producing *Escherichia coli.*

^a^ One probable case.

**Figure 3:**
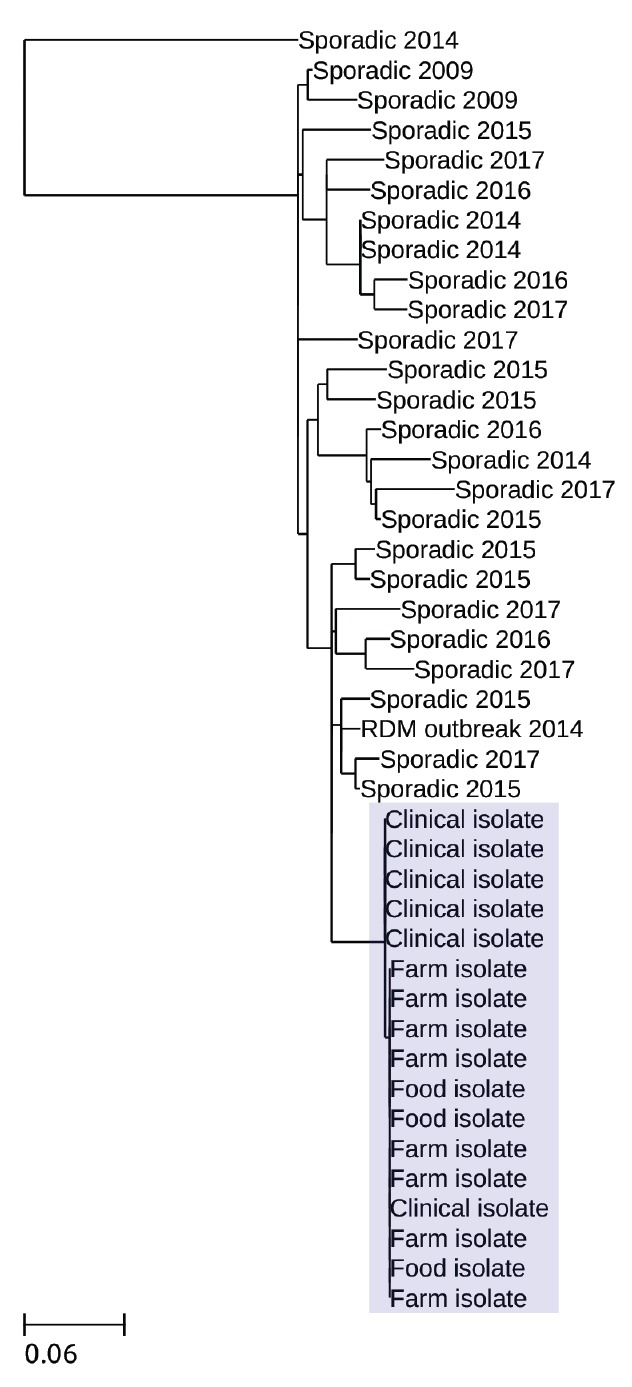
Phylogeny of the clinical, food and farm isolates linked to the STEC O157:H7 outbreak, England, 2017

Five of the seven cases were residents of the Isle of Wight and were notified to PHE’s Hampshire and Isle of Wight Health Protection Team by local clinicians in line with statutory requirements either on detection of presumptive *E. coli* O157 in stool samples at the local hospital or on presentation with HUS. There were two cases resident in a different part of the country; these were both initially identified as likely to be linked to the outbreak through routine review of PHE’s STEC O157 WGS database, which showed isolates from these cases to be genetically identical to the isolate from case 1 (i.e. zero SNP difference between them) (Figure 3). Sequences from other closely related isolates were more than 10 SNPs different from the outbreak strain sequence and were not temporarily related or geographically or epidemiologically linked to the implicated farm.

STEC: Shiga toxin-producing *Escherichia coli.*

Isolates linked to the outbreak are highlighted. The clinical isolates were from the six confirmed cases. Sequences from other closely related sporadic isolates (sporadic isolates defined here as not linked to the outbreak) within the 25 single nucleotide polymorphism (SNP) single linkage cluster available in the Gastrointestinal Bacteria Reference Unit archive are shown for context, including an isolate from an outbreak associated with raw drinking milk in 2014 in the south-west of England .Quality trimmed Illumina reads were mapped to the STEC O157 reference genome Sakai (Genbank accession number: BA000007) using BWA-MEM. SNPs were identified using GATK2 in unified genotyper mode. Core genome positions that had a high quality SNP (> 90% consensus, minimum depth 10×, MQ ≥ 30) in at least one isolate were extracted. SNP positions that were present in at least 80% of isolates were used to derive maximum likelihood phylogenies with RaxML using the GTRCAT model with 1,000 iterations.

Review of STEC ESQs confirmed that all seven cases were either residents of, or had visited, the Isle of Wight within the 7 days before symptom onset. Four cases provided a clear history of consuming RDM from the farm. For a further two cases, there was no clear recollection of whether the cases themselves had consumed the milk. However, both had close relatives who consumed RDM from the farm. One case did not report any epidemiological link to the farm or RDM produced at the farm.

All three cases of HUS had symptom onset after the recall advice was communicated to customers. One case reported that they might have consumed RDM after the recall advice was issued. Another case was unsure if they consumed RDM, but they belonged to a household where RDM was consumed by other family members. The third case reported consuming RDM despite being aware of the recall advice. 

### Environmental investigations

The farm was primarily a dairy farm producing raw and pasteurised milk, cheese and butter. The site included a café, a petting area, an educational centre, holiday accommodation and a shop selling produce from the farm. Overall, the standard of cleanliness at the farm was assessed as very good. It was noted that there was a dedicated car park for visitors with direct access to the café and farm shop. The visitors’ car park and all concrete pathways around the café and farm shop were dry and clean, and no signs of faecal contamination were seen anywhere near these areas. There was no livestock on neighbouring fields and there were no footpaths across the farm land. There was therefore no contact between grazing cattle from the farm and any other livestock, nor potential for the general public to come in contact with grazing cattle. Animal accommodation was assessed as being in a very good state of repair, with spacious and well-ventilated pens for calves and heifers, as well as plenty of bedding. Following the inspection, the installation of further hand washing facilities in the petting area was recommended.

Independent DHI inspections at the farm, with a focus on raw milk production, did not identify any hygiene concerns.

### Microbiological investigations of food and animals samples

STEC O157:H7 PT21/28 was isolated from six of the seven cases linked to this outbreak. Colonies of STEC O157:H7 PT21/28 were detected by PCR from each of the three bulk tank milk samples taken on 25 September 2017. Four subsequent RDM samples from the bulk tank, taken on three separate occasions at least a week apart, were negative. Of note, statutory indicator bacteria tests gave compliant results for all three samples from which STEC O157:H7 was isolated. The standard for RDM is plate count at 30 °C (cfu/mL) < 20,000 and coliforms (cfu/mL) < 100.

No other pathogens were detected in any of the milk samples tested.

Of the animal faecal samples, 13 calf and eight cow samples tested positive for STEC O157:H7. Analysis of the WGS data confirmed that all isolates from the clinical specimens, food and animal samples were different by one SNP (Figure 3).

### Outbreak control measures

The farm management team agreed to voluntarily cease sale of the RDM on 25 September 2017, and to contact customers who had purchased RDM from the farm advising them not to consume the product and to return it. This was in line with the OCT’s advice, along with the recommendation for suspension of animal petting activities until the end of the investigation. Occupational health measures were also put in place to prevent cross-contamination from the farm animals to the food business operations, and to minimise direct and indirect exposure of staff and their families to animal faeces.

Controls on the sale of RDM were maintained throughout the course of the investigation, during which time the DHI issued a formal letter to the farm owner instructing the withdrawal of the raw milk from sale. The FSA Incidents branch provided support to the DHI and coordinated the FSA response. Given the high prevalence of the outbreak strain in animal faecal samples and the likely resources involved in mitigating the risks, the farm decided to voluntarily relinquish their authorisation to sell RDM.

## Discussion

During the outbreak, the rapid detection by PCR of STEC O157:H7 from the RDM samples provided the OCT with robust evidence that RDM was the vehicle of infection, and supported the OCT’s decisions on control measures put in place on the sale and recall of RDM. The enhanced sensitivity of the PCR approach compared to culture alone, in combination with timely sampling of the RDM at the farm, contributed to the successful isolation of the outbreak strain in the contaminated product. The timely sampling of the RDM was initiated following the identification of the epidemiological link early in the course of the investigation.

STEC is notoriously difficult to detect and isolate from food samples, as the infectious dose is low and contamination levels are often below the detection limit of the tests [7]. In recent years, PCR has been used successfully to detect STEC O157:H7 in food samples during outbreaks caused by contaminated watercress [9], cross-contamination at two butchers’ premises [16] and contaminated frozen beef burgers (data not shown), as well as in surveillance studies on RDM [17] and imported curry and banana leaves [18].

Due to restrictions on the sale of RDM that only allow direct sales from the farm to the final consumer (and not via an intermediate retailer), milk-borne outbreaks associated with RDM in the UK are smaller than those caused by pasteurisation failures and, therefore, are more difficult to detect [19]. Although the first two local cases in this outbreak were detected by analysis of the enhanced surveillance data, two further cases—both of whom were resident in a different region of the country—were identified by routine surveillance using WGS data. Consumption of RDM was not reported by all cases; however, in most instances there was evidence that RDM from the farm was available in the home environment and was consumed by members of the household. This highlights a common problem encountered during outbreak investigations, where interviewees fail to recall an accurate food history or may be unaware they consumed the implicated product [7].

A strength of this investigation was the use of STEC WGS surveillance systems to identify and assess cases in England within five SNPs from the designated outbreak profile. WGS is a highly discriminatory typing method that can be used for case ascertainment, regardless of the confounding influence of inaccurate food histories [16,19].

Previous studies have shown that different Stx subtypes are associated with varying clinical outcomes, with Stx2a most likely to be associated with severe disease [20]. Although PCR was available for identification of the different Stx subtypes before the implementation of WGS, the assay was labour intensive and the results were difficult to interpret; as a result, the PCR assays were not used routinely at PHE. Since the implementation of WGS, the Stx subtype has been routinely available as part of the downstream bioinformatics pipeline [15]. The strain associated with this outbreak had *stx2a*, and the OCT was informed at the beginning of the investigation that it was highly likely to cause severe disease, including HUS. This information contributed to the risk management of the outbreak, providing further evidence that suspension of the sale of RDM and animal petting activities, as well as the product recall, were justified. The timely product recall advice undoubtedly prevented further cases.

In England, Wales and Northern Ireland, farms may sell RDM directly to the consumer on the farm, in a farmhouse catering operation, via door-to-door delivery service, through the Internet or at farmers’ markets [21]. Restrictions on the sale of RDM are governed by the Food Hygiene (England) Regulations (2006) [22]. In a recent study of RDM carried out by PHE between 2014 and 2016, 454 of 770 (59.0%) samples of RDM collected from retail sales in England for routine monitoring were of a satisfactory quality [17].

Between 2012 and 2017, the volume of RDM produced in the UK has increased fivefold [23]; during this period, there has also been an increase in gastrointestinal outbreaks associated with the consumption of unpasteurised dairy products, several having been reported since 2014 [23,24]. Prior to 2014, the last outbreaks associated with RDM in the UK occurred in 2002 [23].

In this outbreak, five of the seven cases were children. Severe symptoms of gastrointestinal disease caused by STEC O157 are seen more frequently in younger children [1]. It is of concern that the families were not aware of the risk or, if they were, that they felt the risk was acceptable. Consumers of RDM may be influenced by information promoting the perceived benefits of RDM, without balancing this against the risk of food-borne infection [25]; this situation highlights the issue of risk perception more generally [26]. The FSA is currently working to improve the risk communication for RDM, including introducing revised labels with clearer warnings for vulnerable groups, including children and people with underlying health conditions [23].

The farm implicated in this outbreak was classed as small, with only cattle farmed. The farm had produced RDM for several decades, and no previous incidents had been recorded. During the outbreak investigation, no changes to the manufacturing protocols were identified. However, it was established that although the farm operation had always been ‘closed’, i.e. with no acquisition of livestock from other farms, 4 months before the outbreak a small number of animals were brought onto the farm from a neighbouring farm. Although they were accommodated in a separate area with reportedly no mixing, it is possible that there may have been indirect cross-contamination and that the outbreak strain was introduced into the indigenous herd from these visiting animals.

### Conclusions

The outbreak investigation described here demonstrates how recent advances in laboratory technology, specifically PCR for the detection of STEC in food and WGS for case ascertainment and highly discriminatory strain typing, can inform outbreak detection and investigation. Successful integration of traditional epidemiological surveillance and advanced laboratory methods for characterisation of human, animal and environmental samples enabled identification of the outbreak vehicle, leading to implementation of effective control measures in a timely manner.
